# Health systems readiness and quality of inpatient malaria case-management in Kano State, Nigeria

**DOI:** 10.1186/s12936-020-03449-5

**Published:** 2020-10-30

**Authors:** Abiodun A. Ojo, Kolawole Maxwell, Olusola Oresanya, Justice Adaji, Prudence Hamade, James K. Tibenderana, Saddiq S. Abubakar, Bala M. Audu, Ahmad Njidda, Aishatu B. Gubio, Robert W. Snow, Dejan Zurovac

**Affiliations:** 1 Malaria Consortium, Abuja, Nigeria; 2 Malaria Consortium, London, UK; 3 State Malaria Elimination Programme, Kano, Nigeria; 4 National Malaria Elimination Programme, Abuja, Nigeria; 5 KEMRI-Wellcome Trust Research Programme, Nairobi, Kenya; 6 Centre for Tropical Medicine and Global Health, University of Oxford, Oxford, UK

**Keywords:** Malaria, Hospital, Systems readiness, Case-management, Test and treat, Artesunate, Nigeria

## Abstract

**Background:**

Nigeria was among the first African countries to adopt and implement change of treatment policy for severe malaria from quinine to artesunate. Seven years after the policy change health systems readiness and quality of inpatient malaria case-management practices were evaluated in Kano State of Nigeria.

**Methods:**

A cross-sectional survey was undertaken in May 2019 at all public hospitals. Data collection comprised hospital assessments, interviews with inpatient health workers and data extraction from medical files for all suspected malaria patients admitted to the paediatric and medical wards in April 2019. Descriptive analyses included 22 hospitals, 154 health workers and 1,807 suspected malaria admissions analysed from malaria test and treat case-management perspective.

**Results:**

73% of hospitals provided malaria microscopy, 27% had rapid diagnostic tests and 23% were unable to perform any parasitological malaria diagnosis. Artemisinin-based combination therapy (ACT) was available at 96% of hospitals, artemether vials at 68% while injectable quinine and artesunate were equally stocked at 59% of hospitals. 32%, 21% and 15% of health workers had been exposed to relevant trainings, guidelines and supervision respectively. 47% of suspected malaria patients were tested while repeat testing was rare (7%). 60% of confirmed severe malaria patients were prescribed artesunate. Only 4% of admitted non-severe test positive cases were treated with ACT, while 76% of test negative patients were prescribed an anti-malarial. Artemether was the most common anti-malarial treatment for non-severe test positive (55%), test negative (43%) and patients not tested for malaria (45%). In all categories of the patients, except for confirmed severe cases, artemether was more commonly prescribed for adults compared to children. 44% of artesunate-treated patients were prescribed ACT follow-on treatment. Overall compliance with test and treat policy for malaria was 13%.

**Conclusions:**

Translation of new treatment policy for severe malaria into inpatient practice is compromised by lack of malaria diagnostics, stock-outs of artesunate and suboptimal health workers’ practices. Establishment of the effective supply chain and on-going supportive interventions for health workers accompanied with regular monitoring of the systems readiness and clinical practices are urgently needed.

## Background

Nigeria contributes 25% of the global malaria mortality burden [1]. Malaria comprises 30% of admissions in Nigerian hospitals [1, 2]. Nigeria was among the first African countries to adopt the 2012 World Health Organization (WHO) recommended artesunate treatment policy for severe malaria and implement a series of programmatic activities to facilitate readiness of hospitals and health workers to deliver new recommendations countrywide [2–4]. There is, however, limited evidence from Nigerian hospitals about the systems readiness for the new case-management standards and actual care delivered to the patients admitted with suspected malaria [5]. Such deficiencies are not unique to Nigeria—there is a generally dearth of studies evaluating compliance with inpatient malaria guidelines across Africa [6–12], only a few have examined this topic beyond the paediatric population [6, 10, 12] and hardly any after the artesunate policy has been implemented [11, 12]. Despite the differences in scope, scale, context and methodological aspects of these studies, most have however reported deficiencies related to the suboptimal coverage of interventions and common practices in discordance with national guidelines. In this manuscript, an evaluation from Kano state, Northern Nigeria where hospital and health worker readiness for the policy implementation, health worker knowledge about case-management recommendations, and the quality of inpatient management for patients admitted with suspected malaria has been evaluated seven years after the change of treatment policy from parenteral quinine to artesunate, is reported.

## Methods

### Study area and context

**Fig. 1:**
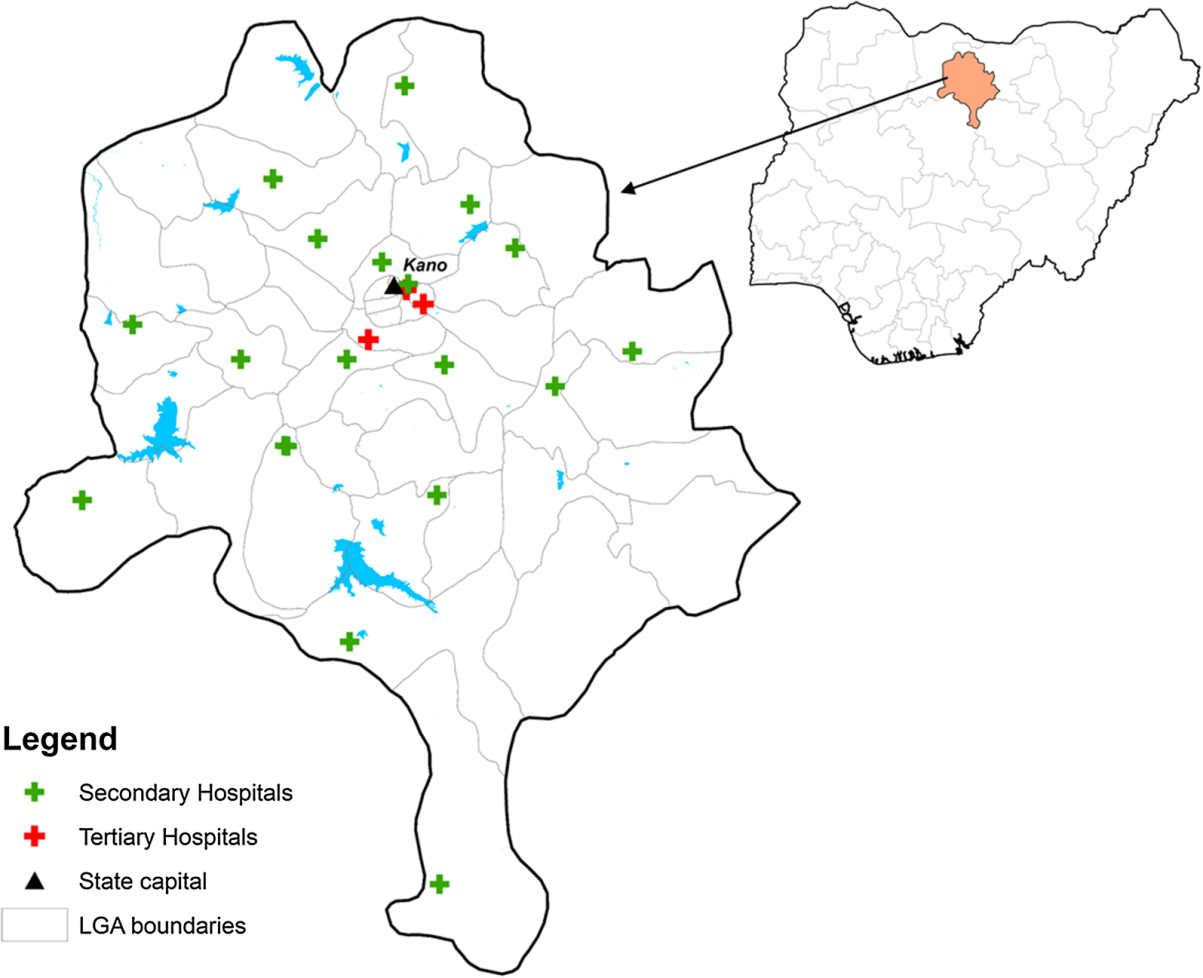
Map of study area and survey hospitals within Kano state and Nigeria

Kano State is situated in the Sahelian region in the North-West zone of Nigeria (Fig. 1). It is the second most populous state with an estimated population of 13 million. The state consists primarily of Sudan savanna vegetation with a wet season occurring between May and September. Malaria transmission is endemic with seasonal peaks between September and February. Predicted community-based *Plasmodium falciparum* prevalence rates in children aged 2–10 years were estimated at 33% in 2015 [13]. Of relevance for inpatient malaria management, the Federal and State governments have been working with donors and implementing partners since 2012 to support the procurement and distribution of injectable artesunate, oral artesunate-based combination therapy (ACT) and rapid diagnostic tests (RDT). In addition, there has been a concerted effort to develop and distribute national guidelines and treatment job-aids, deliver in-service trainings of the front-line health workers on the management of severe malaria and artesunate use, support training of malaria microscopists, and implement integrated supportive supervision including on job capacity building of the hospital health workers [14, 15]. The policy of free of charge diagnosis and treatment for the donor and government procured RDTs and medicines for malaria is in place in all public hospitals.

### Study design and data collection

A cross-sectional survey was undertaken at all public hospitals in Kano state in May 2019. Hospitals providing specialized services (e.g. orthopedic, psychiatric), those admitting only special patient groups (e.g. military, pregnant women) and those without functional paediatric or medical wards were excluded. Survey teams, comprised of one hospital records officer and one hospital nurse, were trained over three days to collect data. Three methods of data collection were followed: (1) hospital assessments; (2) interviews and knowledge assessments of the inpatient health workers; and (3) data extraction from the paediatric and medical ward admission files archived at the hospital medical records office. On the first day of data collection, the physical assessments of the availability of anti-malarial medicines and RDTs, displayed job aids in the study wards, and the availability of malaria related laboratory services with focus on malaria microscopy was established at relevant hospital departments. All clinicians and qualified nurses on the day shift duty in the paediatric and medical wards on the first day of data collection were approached and, upon providing written informed consent, interviewed by the study nurse. The information about health worker demographics, exposure to relevant in-service trainings, case-management guidelines, supportive supervision, and their knowledge about management of severe malaria and artesunate use was collected. The knowledge was mainly assessed using self-administered, multiple choice questions with single correct responses provided to the health workers after the assessments. With respect to data extraction method, ward admission registers were used to list all patients admitted to the paediatric and medical wards for April 2019. Admission files were then retrieved from the records office and screened to determine those reflecting patients with suspected malaria criteria on admission, defined as documentation of the complaint of fever or history of fever, temperature ≥ 37.5 °C, diagnosis or impression of malaria, or prescription of any anti-malarial treatment. Among patients with suspected malaria, data were extracted including age, sex, weight, dates of admission and discharge, assessments and laboratory tests performed with results recorded, diagnoses made, and anti-malarial treatments prescribed during the hospital stay and upon discharge. All patients having indication of malaria test ordered and no test result in the admission file were cross-checked in the laboratory register to determine whether malaria test was performed and what was the test result.

### Indicators and analysis

The main indicators were constructed to reflect health systems readiness and test and treat case-management standards for the management of patients admitted with suspected malaria [4]. The readiness indicators at the hospital and health worker level referred to the coverage of hospitals and inpatient health workers with interventions relevant for the management of malaria such as availability of anti-malarials, malaria diagnostics, laboratory support, among others, and the coverage of health workers with relevant in-service trainings, guidelines and supportive supervision including levels of knowledge about recommended standards. The test and treat case-management indicators reflected compliance with national guidelines recommending that all patients admitted with suspected malaria should be tested for malaria and depending on the malaria severity criteria and test result should have either parenteral artesunate prescribed for severe test positive patients (confirmed severe malaria), ACT prescribed for non-severe test positive patients or no anti-malarial prescribed for test negative patients [4]. Severity features among suspected malaria admissions were determined as either documentation of any of the guideline recommended criteria on admission or, recognizing documentation limitations of the clinical criteria, by health workers diagnosis of severe malaria made on admission (Table 1).

**Table 1:** Documented severity criteria and malaria admission diagnoses, by admission ward

	Paediatric ward (N = 891)	Medical ward (N = 858)	All admissions (N = 1749)
	n (%)	n (%)	n (%)
Clinical or laboratory features			
Severe anaemia^a^	86 (9.7)	96 (11.2)	182 (10.4)
Convulsions^b^	85 (9.5)	26 (3.0)	111 (6.4)
Persistent vomiting	62 (7.0)	58 (6.8)	120 (6.9)
Prostration^c^	39 (4.4)	56 (6.5)	95 (5.4)
Shock^d^	11 (1.2)	42 (4.9)	53 (3.0)
Respiratory distress^e^	16 (1.8)	25 (2.9)	41 (2.3)
Impaired consciousness^f^	10 (1.1)	24 (2.8)	34 (1.9)
Jaundice	9 (1.0)	16 (1.9)	25 (1.4)
Hypoglycaemia^g^	3 (0.3)	6 (0.7)	9 (0.5)
Haemoglobinuria^h^	2 (0.2)	6 (0.7)	8 (0.5)
Renal failure^i^	4 (0.5)	3 (0.4)	7 (0.4)
Pulmonary oedema	2 (0.2)	5 (0.6)	7 (0.4)
Abnormal bleeding	0	3 (0.4)	3 (0.2)
Any clinical or laboratory features	248 (27.8)	245 (28.6)	493 (28.2)
Malaria admission diagnoses			
“Malaria” (unclassified)	289 (32.4)	280 (32.6)	569 (32.5)
Any form of severe malaria diagnosis	112 (12.6)	86 (10.0)	198 (11.3)
Severe malaria	104 (11.7)	75 (8.7)	179 (10.2)
Cerebral malaria	3 (0.3)	9 (1.1)	12 (0.7)
Complicated malaria	7 (0.8)	7 (0.8)	14 (0.8)
Uncomplicated/non-severe	4 (0.5)	12 (1.4)	16 (0.9)
Any study severe malaria criteria	285 (33.2)	305 (34.2)	590 (33.7)

^a^“Hb < 5 g/dl or HCT < 15% in children < 12yrs (< 7 g/dl and < 20% respectively in > 12yrs)”

^b^“Convulsions, fits or seizures “

^c^“Unable to drink/breastfeed/sit/stand/walk or prostrated”

^d^“Capillary refill >  = 3 s, systolic BP < 80 mmHg in adults or < 70 mmHg in children or “shock”

^e^“Acidotic/deep breathing, chest in-drawing or respiratory distress”,

^f^“Drowsiness, lethargy, confusion, unconsciousness, coma or GCS < 15/AVPU < A”

^g^“Blood sugar < 2.2 mmol/l or < 40 mg/dL”

^h^“Dark urine, blood in urine, haematuria”

^i^“Oliguria, anuria or renal failure”

Therefore, four categories of patients were determined to assess malaria treatment practices. The first category included severe malaria patients defined as the presence of positive malaria test on admission, and either documentation of any feature of severe malaria or presence of severe malaria diagnosis made by clinician. The remaining three categories comprised test positive non-severe malaria patients, test negative patients with or without severity criteria; and finally, patients not tested for malaria regardless of clinical features. Finally, further patient level indicators referring to the performance of the repeat testing, follow-on treatments, and artesunate dosing were also measured.

The analysis of indicators was undertaken at hospital, health worker and patient levels. Correctness of case-management was analysed from malaria test and treat perspective without considering management of comorbidities and severe malaria complications. Primary analyses included all assessed hospitals, all interviewed health workers and all suspected malaria patients or their subcategories evaluated for the case-management practices. Analyses stratified by ward (paediatric *vs* combined male and female medical) and health workers cadre (clinicians *vs* nurses) were also undertaken on several indicators. Descriptive statistics formed the basis of analysis through frequencies, means and medians and inter-quartile ranges for non-normally distributed data. Data entry, coding, cleaning, and analysis was undertaken using Access (Microsoft, USA) and STATA, version 14 (StataCorp, USA).

## Results

### Description of study populations

The survey comprised 22 assessed hospitals, 154 interviewed inpatient health workers, and 3,084 medical files reviewed for patients admitted to the paediatric and medical wards in April 2019. Of 154 health workers, the majority were male (57.1%), had a median age of 35 years [IQR: 28–43] and 5 years of inpatient experience [IQR: 3–15]. Most health workers were nurses (99; 64.3%) while of 55 clinicians, about half were medical officers (29; 52.7%) followed by house officers (14; 24.5%), resident doctors (11; 20.0%) and consultants (1; 1.8%). Two-thirds (65.6%) of health workers were on duty in medical wards and about a third (34.4%) in the paediatric ward.

**Fig. 2:**
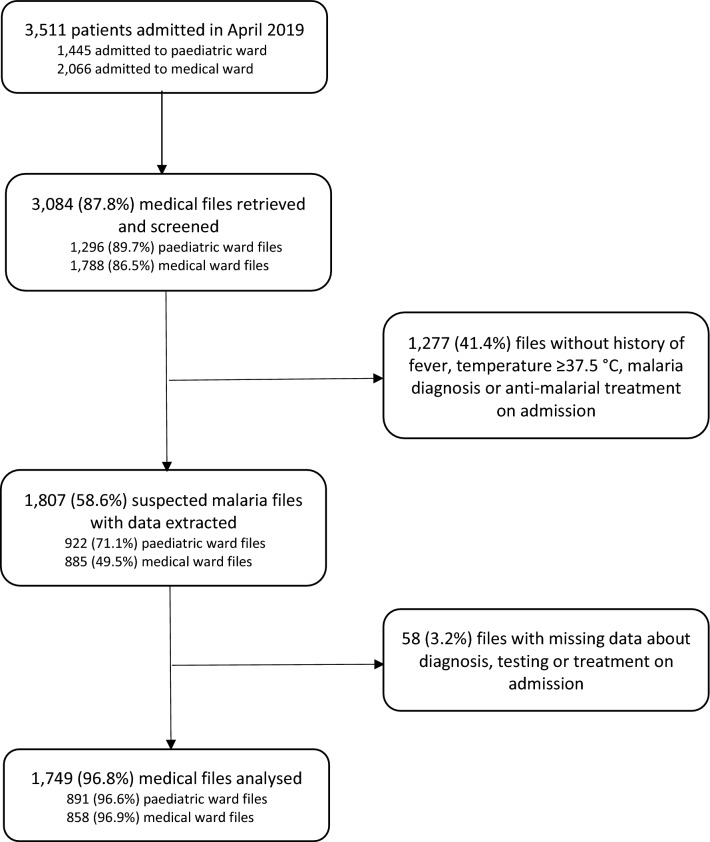
Patients’ records retrieved, screened and analysed, by admission ward

Of 3,511 patients admitted in the study period, the medical files were retrieved for 3,084 patients (87.8% success rate) (Fig. 2). Data were extracted for 1,807 (58.6%) patients who met criteria of suspected malaria, more commonly for children in the paediatric (71.1%) than for patients in medical wards (49.5%). Fifty-eight patients (3.2%) meeting screening criteria were excluded from analysis due to incomplete data. Finally, 1,749 suspected malaria admissions were analysed. The overall patients’ gender distribution was similar (50.3% vs 49.6%) though females were more represented among adults in medical wards (55.2% vs 44.8%) while male children were more commonly admitted to the pediatric ward (55.7% 44.3%). The median age of paediatric and medical ward patients was respectively 2 [IQR: 1–4] and 38 [IQR: 25–59] years. The median length of admission was 2 [IQR: 1–4] days in the paediatric and 3 [IQR: 2–5] days in medical wards, as measured by number of nights spent at hospitals.

Among all suspected malaria patients, the most commonly documented malaria severity features included severe anaemia (10.4%), persistent vomiting (6.9%), convulsions (6.4%) and prostration (5.4%) while the remaining features were documented in less than 5% of the patients (Table 1). At least one clinical feature of malaria severity was documented in 28.2% of patients, similarly among children (27.8%) and adults (28.6%). Complementing severity features with routine diagnosis of either severe, complicated or cerebral malaria on admission, 33.7% of suspected malaria admissions met study criteria of severe malaria. Table 1 shows documented severity features and malaria admission diagnoses stratified by admission ward.

### Hospital readiness for inpatient malaria case-management

Of 22 hospitals assessed, nearly three-quarters (73%) provided malaria microscopy on assessment days, 27% stocked non-expired RDTs, and despite RDTs complementing diagnostic services, 23% of hospital laboratories were unable to provide at least one parasitological malaria diagnostic service (Table 2). At the laboratories providing microscopy, blood smear preparation using thick and thin smear was rarely performed (20%), 56% of the laboratories stained slides using Giemsa solution, 44% performed parasite differentiation, and finally none of the laboratories reported parasite densities using recommended counting methods per ml/WBC.

**Table 2:** Hospital readiness for implementation of inpatient malaria test and treat policy, Kano state

Hospital characteristics (N = 22)	n (%)
Availability of malaria diagnostic services	
Any malaria diagnostics (RDT or microscopy)	17 (77)
Functional malaria microscopy	16 (73)
Non-expired RDTs in stock	6 (27)
Availability of anti-malarials	
Any injectable anti-malarial drug in stock on survey days	19 (86)
Artesunate injections	13 (59)
Artemether injections	15 (68)
Quinine injections	13 (59)
Any ACT in stock on survey days	21 (96)
Artemether-lumefantrine tablets	20 (91)
Artesunate-amodiaquine tablets	19 (86)
Artesunate stock-out experienced in past 3 months	8 (37)
Availability of any malaria diagnostics, artesunate and any ACT	10 (46)
Availability of job aids in admission wards^a^	
Artesunate poster displayed in at least one ward	7 (32)
Artesunate poster displayed in all wards	4 (18)
ACT dosing poster displayed in at least one ward	2 (9)

^a^Paediatric, female or male medical wards

Pharmacy assessments revealed that artesunate and quinine were equally available at 59% of hospitals, less commonly than injectable artemether (68%). About 14% of hospitals were without injectable anti-malarials during the assessment days. With respect to ACT, the availability of oral preparations of artemether-lumefantrine (AL) or artesunate-amodiaquine (AA) was higher than injectable anti-malarials, most hospitals had both ACT medicines in stock (19/22), and all except one hospital stocked at least one of the recommended artemisnin-based combinations.

Furthermore, 68% of hospitals had quinine tablets in stock, 14% had artesunate suppositories and only one hospital was found with expired artesunate vials. Artesunate stock-outs were also reported by 37% of hospitals during the 3-month period prior to the survey. Finally, a third of the hospitals (32%) had at least one ward with displayed artesunate administration poster while at only 9% of hospitals ACT dosing poster was observed. Overall, only 46% of hospitals had on survey days the basic capacity to test for malaria and treat with artesunate and ACT (Table 2).

### Health worker readiness for inpatient malaria case-management

Health worker readiness for inpatient malaria management, comprised of their exposure to the programmatic interventions and their knowledge about malaria diagnosis and treatment recommendations, was assessed among clinicians and nurses from the paediatric and medical wards (Tables 3 and 4). About a third of health workers (32.2%) received training on severe malaria management and artesunate use, 21.0% had access to the latest national malaria case-management guidelines while the exposure to any supportive supervision and the supervision on severe malaria management was low (23.6% and 14.6%, respectively). Clinicians compared to nurses were more commonly trained on severe malaria (39.6% *vs* 28.4%) and more often had guidelines (35.3% *vs* 13.4%). The exposure to the supervision was however similarly low between the cadres (13.7% *vs* 15.1%). Most health workers knew that all admitted patients with fever should be tested for malaria (79.7%), that children and non-pregnant adults with severe malaria should be treated with artesunate (85.5%), that minimum of three artesunate doses should be prescribed (87.0%) and that artesunate should be followed on with ACT as soon as the patient can take oral therapy (78.3%). The lack of artesunate treatment knowledge was however evident for pregnant women with severe malaria in the first (46.4%) and in later trimesters of the pregnancy (68.8%). The knowledge gaps were also observed with respect to artesunate dosing recommendations, both for children weighing < 20 kg where 3.0 mg/kg is recommended (53.6%) and for patients over 20 kg with 2.4 mg/kg recommendations (60.9%). Overall, less than half of health workers (45.7%) had correct dosing knowledge for both weight groups. Finally, the knowledge about all testing, treatment and dosing recommendations was higher among clinicians than nurses (Table 4).

**Table 3:** Health workers’ exposure to the programmatic support interventions in Kano state, by cadre

Support interventions	Clinicians (N = 55)	Nurses (N = 99)	All HWs (N = 154)
	n (%)	n (%)	n (%)
Training^a^			
Trained on any malaria case-management	21 (43.8)	30 (31.6)	51 (35.7)
Trained on severe malaria case-management	19 (39.6)	29 (30.5)	48 (33.6)
Trained on artesunate use	21 (43.8)	27 (28.4)	48 (33.6)
Trained on severe malaria case-management and artesunate use	19 (39.6)	27 (28.4)	46 (32.2)
Guidelines^a^			
Has national malaria case-management guideline	18 (35.3)	13 (13.4)	31 (21.0)
Supportive supervision^a^			
Any supervisory visit in last 3 months	11 (21.6)	23 (24.7)	34 (23.6)
Supervision on severe malaria case-management	7 (13.7)	14 (15.1)	21 (14.6)
Supervision on artesunate use	6 (11.8)	14 (15.1)	20 (13.9)

^a^Denominators exclude health workers without complete intervention data set, respectively 11, 5 and 10 observations for the training, guidelines and supervision intervention

**Table 4:** Knowledge about malaria diagnosis and treatment recommendations in Kano state, by cadre

Health workers knowledge [correct responses]	Clinicians (N = 48)	Nurses (N = 90)	All HWs (N = 138)
	n (%)	n (%)	n (%)
Malaria testing [all fevers]	41 (85.4)	69 (76.7)	110 (79.7)
Treatment for severe malaria			
1) Children and adults [AS]	44 (91.7)	74 (82.2)	118 (85.5)
2) First trimester pregnancy [AS]	25 (52.1)	39 (43.3)	64 (46.4)
3) 2nd and 3rd trimester pregnancy [AS]	39 (81.3)	56 (62.2)	95 (68.8)
4) Follow-on treatment [AL or AA]	45 (93.8)	63 (70.0)	108 (78.3)
AS reconstitution [bicarbonate]	40 (83.3)	76 (84.4)	116 (84.1)
AS dilution [saline or 5% dextrose]	29 (60.4)	44 (48.9)	73 (52.9)
AS dosing knowledge < 20 kg [3 mg/kg]	28 (58.3)	46 (51.1)	74 (53.6)
AS dosing knowledge > 20 kg [2.4 mg/kg]	39 (81.3)	45 (50.0)	84 (60.9)
Correct dose for both weight groups	26 (54.2)	37 (41.1)	63 (45.7)
Minimum number of AS doses [3]	43 (89.6)	77 (85.6)	120 (87.0)
AS dosing interval [0, 12, 24, 48]	31 (64.6)	34 (37.8)	65 (47.1)

### Malaria case-management by admission ward

Table 5 shows main inpatient test and treat malaria case-management practices stratified by admission ward. Overall test and treat performance in the management of 1,749 suspected malaria cases was 13.2% with similar quality levels observed for paediatric (14.3%) and medical ward (12.1%) patients. Stratified analyses by the performance of malaria testing and treatment prescribed based on test results and severity criteria reveals details of malaria case-management practices. First, testing of suspected malaria patients was low on admission (46.8%), both in children (49.6%) and adults (43.8%). When malaria tests were performed, most patients were tested using malaria microscopy (56.4%). Repeat testing was rare (7.6%). Second, artesunate treatment for confirmed severe malaria patients was suboptimal (59.6%) and nearly equal for paediatric (59.9%) and medical ward patients (59.2%). Nearly all patients (97.9%) were prescribed intravenous route of artesunate. While quinine use was negligible (0.2%) parenteral artemether was prescribed for 23.1% of confirmed severe malaria cases, similarly for children (22.2%) and adults (24.2%). Third, test positive patients without severe malaria criteria (neither clinically documented nor diagnosed as severe malaria by clinicians) were rarely treated with recommended oral ACT (4.4%). Parenteral anti-malarial therapy for this group of patients was nearly universal (95.6%), however, in contrast to severe test positive patents, with major prescribing shift from artesunate (31.7%) to artemether (49.1%). Fourth, compliance with no anti-malarial recommendations for test negative patients was low (24.2%). As similarly observed for test positive non-severe cases most of the test negative patients were treated with parenteral anti-malarials (70.2%) and more commonly with artemether (43.4%) than artesunate (17.7%). In both categories of patients (test negative and test positive non-severe patients) parenteral artemether (or arteether) was more commonly prescribed for adults compared to children (54.6% *vs* 37.7% and 63.7% *vs* 49.7%, respectively). Finally, among patients who were not tested for malaria most were prescribed anti-malarials (87.8%) with artemether being most commonly prescribed (44.8%), particularly among adults (51.1%) (Table 5).

**Table 5:** Quality of inpatient malaria case-management in Kano state, by admission ward

	Paediatric ward (N = 891)	Medical ward (N = 858)	All patients (N = 1,749)
	n (%)	n (%)	n (%)
Composite test and treat performance^a^	127 (14.3)	104 (12.1)	231 (13.2)
Malaria test performed	442 (49.6)	376 (43.8)	818 (46.8)
Malaria test repeated	40 (9.1)	22 (5.9)	62 (7.6)
Treatment for test positive severe cases	N = 162	N = 120	N = 282
Artesunate parenteral	97 (59.9)	71 (59.2)	168 (59.6)
Artemether parenteral	36 (22.2)	29 (24.2)	65 (23.1)
Arteether parenteral	4 (2.5)	6 (5.0)	10 (3.6)
ACT	13 (8.0)	8 (6.7)	21 (7.5)
Quinine parenteral	3 (1.9)	1 (0.8)	4 (0.2)
Other anti-malarial treatments^b^	9 (5.6)	5 (4.2)	14 (5.0)
Treatment for test positive non-severe cases	N = 203	N = 135	N = 338
ACT	10 (4.9)	5 (3.7)	15 (4.4)
Artesunate parenteral	71 (35.0)	36 (26.7)	107 (31.7)
Artemether parenteral	92 (45.3)	74 (54.8)	166 (49.1)
Arteether parenteral	9 (4.4)	12 (8.9)	21 (6.2)
Quinine parenteral	5 (2.5)	3 (2.2)	8 (2.4)
Other anti-malarial treatments^c^	16 (7.9)	5 (3.7)	21 (6.2)
Treatment for test negative cases	N = 77	N = 121	N = 198
No anti-malarial treatment	20 (26.0)	28 (23.1)	48 (24.2)
Artesunate parenteral	16 (20.8)	19 (15.7)	35 (17.7)
Artemether parenteral	26 (33.8)	60 (49.6)	86 (43.4)
Arteether parenteral	3 (3.9)	6 (5.0)	9 (4.6)
ACT	8 (10.4)	3 (2.5)	11 (5.6)
Quinine parenteral	0	1 (0.8)	1 (0.5)
Other anti-malarial treatments^d^	4 (5.2)	4 (3.3)	8 (4.5)
Treatment for not tested patients	N = 449	N = 482	N = 931
No anti-malarial treatment	52 (11.6)	62 (12.9)	114 (12.2)
Artesunate parenteral	159 (35.4)	81 (16.8)	240 (25.8)
Artemether parenteral	171 (38.1)	246 (51.0)	417 (44.8)
Arteether parenteral	15 (3.3)	19 (3.9)	34 (3.7)
ACT	31 (6.9)	49 (10.2)	80 (8.6)
Quinine parenteral	3 (0.7)	4 (0.8)	7 (0.8)
Other anti-malarial treatments^e^	18 (4.0)	21 (4.4)	39 (4.2)

^a^Performance of malaria test and either artesunate treatment for test positive severe cases, ACT for test positive non-severe cases or no anti-malarial treatment for test negative cases

^b^Include 11 AS/artemether, 1 arteether/artemether, 1 AS/quinine and 1 AS/arteether treatment

^c^Include 11 AS/artemether, 3 arteether/artemether, 2 AS/quinine, 2 artemether/quinine, 1 arteether/quinine, 1 AS/arteether and 1 SP treatment

^d^Include 4 AS/artemether and 4 arteether/artemether treatment

^e^Include 15 AS/artemether, 13 arteether/artemether, 3 AS/arteether, 3 SP, 1 quinine/artemether, 1 AS/quinine, 1 artemether syrup, 1 ACT/SP and 1 quinine/artemether/arteether treatment

### Artesunate prescribing and ACT follow-on treatment practices

Of 550 patients treated with parenteral artesunate dosing and ACT follow-on treatment practices were analysed for 431 (78.4%) patients who had specified dose and number of doses prescribed (Table 6). Nearly all patients were prescribed at least three doses of artesunate (97.0%), similarly for children (96.9%) and adults (97.1%). Less than half of the patients (43.9%) were prescribed ACT follow-on treatment, both for children (42.9%) and adults (45.3%). Only 43.4% of patients had complete treatment composed of three or more artesunate doses and ACT prescribed. Weight was measured for 23.7% of all artesunate prescribed patients, for 31.8% of children in the paediatric ward, and for only 11.2% of adults in medical wards. Finally, while lack of weighing precluded measurements of the dose correctness, artesunate dosing was however strikingly uniformed with 80.6% of adults prescribed 120 mg in medical wards and 60.9% of children prescribed either 30 or 60 mg in the paediatric ward.

**Table 6:** Artesunate and follow-on treatment practices in Kano state, by admission ward

Artesunate prescribed patients	Paediatric ward (N = 261)	Medical ward (N = 170)	All patients (N = 431)
	n (%)	n (%)	n (%)
Weight measured	83 (31.8)	19 (11.2)	102 (23.7)
Artesunate 3 or more doses	253 (96.9)	165 (97.1)	418 (97.0)
ACT follow-on treatment	112 (42.9)	77 (45.3)	189 (43.9)
Complete treatment prescribed	110 (42.2)	77 (45.3)	187 (43.4)

## Discussion

The health systems readiness and test and treat based inpatient malaria case-management was evaluated seven years after the change of treatment policy for severe malaria from quinine to artesunate in Nigeria. The findings revealed several strengths but also major readiness and clinical practice deficiencies which severely compromise the quality of service delivery for patients admitted with malaria in Kano State.

The absence of parasitological diagnostic capacities from nearly a quarter of hospitals preclude implementation of universal test and treat policy for malaria. During the past decade lack of malaria diagnostics has been reported from the peripheral facilities across Africa [16, 17] however this has not been common finding at the hospitals where malaria microscopy has been the traditional mainstay of the laboratory services [12, 18]. Furthermore, when malaria microscopy is provided at the study hospitals, the basic microscopy practices such as smear preparations, staining, parasite differentiation and density reporting were largely in discordance with national standards [4]. Introduction of the quality assurance system for malaria microscopy [19], prioritization of microscopy for inpatient management, and increased hospital availability of malaria RDTs focusing on outpatient malaria screening should be the programmatic and organizational priorities targeting improved diagnostic services in the study area.

Seven years after the change of treatment policy from quinine to artesunate, the availability of artesunate was not higher compared to quinine and it was indeed lower compared to injectable artemether. The study did not investigate the supply chain, however, anecdotal reports suggest that poor quantification, loss of medicines during distributions, and central artesunate stock-outs result in insufficient artesunate supplies, local procurements of less expensive medicines, and subsequently compromised implementation of the most effective and policy free treatment for severe malaria. While not unique to Nigeria [12], artesunate stock-outs experienced by over a third of the hospitals and artesunate administration posters displayed in less than a third of hospitals to support its use highlight further policy implementation gaps. Moreover, while delivery of the treatment should never be precluded, conditional recommendations on the use of artemether and quinine treatments in absence of artesunate may also create implementation ambiguities which do not facilitate delivery of the optimum treatment [3, 4]. More positively, nearly universal availability of the free ACT does ensure hospital readiness for the treatment of uncomplicated malaria as well as the readiness for the follow-on treatment of severe malaria. Resolution of artesunate stock-outs, distribution of artesunate job-aids and maintenance of the supply chain for ACT medicines is the major supply chain priority for anti-malarial commodities in the study area.

Despite low coverage of health workers with in-service training, guidelines and supervision, their knowledge about key testing and treatment recommendations for malaria was high. Most health workers, especially clinicians, were aware that all patients admitted with fever should be tested for malaria, that artesunate is the recommended treatment for severe malaria, that minimum of three artesunate doses should be prescribed, and that follow-on treatment for severe malaria is oral ACT. Lower knowledge observed among nurses are the findings likely reflecting non-prescribing role of the nurses in the inpatient setting. Yet, major knowledge gaps calling for supportive interventions are found with respect to the treatment of malaria in pregnancy and artesunate dosing (especially for small children), the deficiency similarly shown in Kenya [12, 20], Tanzania [21] and in other countries across Africa regarding the management of pregnant women with uncomplicated malaria [22].

Despite relatively high levels of knowledge health workers’ compliance with test and treat recommendations for malaria was low, both for the patients admitted to paediatric and medical wards. Testing of less than half of the suspected malaria patients on admission and negligible levels of the repeat testing is the performance lower than reported in several east African countries [7–12]. Absence of diagnostic services, high laboratory workloads, malaria microscopy charges, stock-outs of the policy free RDTs but also clinicians’ behavioral aspects of non-equalizing of all fevers with suspected malaria and testing ignorance due to high test positivity rates (> 80% in children) are some of the possible explanations.

With respect to anti-malarial treatments, two encouraging findings were observed. First, despite high availability of injectable quinine at the hospitals, its use has been nearly discontinued. Second, efforts in deploying new treatment policy were observed for patients with confirmed severe malaria and majority of these patients are indeed prescribed artesunate. Less encouraging, test positive non-severe patients, deserving treatment with oral ACT, are treated with injectable anti-malarials. Similarly, irrational use of anti-malarials was notable among test negative patients. Both deficiencies, at different scales, have been observed among different inpatient populations in various hospital settings across Africa [7–12, 23]. Interestingly, for both categories of the patients, including those who are not tested for malaria, the choice of either artemether or arteether was more common treatment selection than artesunate use. Furthermore, for the same categories of the patients, injectable anti-malarials other than artesunate were more commonly prescribed for adults compared to children, the results in contrast with confirmed severe malaria cases where equal proportion of 60% of children and adults are treated with artesunate. The observed treatment patterns are likely reflection of the insufficient supplies of artesunate, local procurement of medicines, higher cost of artesunate compared to other anti-malarials, and rationing of artesunate use for those patients who have higher likelihood of the treatment indication (e.g. severe confirmed cases), who are at higher mortality risk and whose treatment is less costly such as children. While such practices in the imperfect health system may be reasonable, behavioural deficiencies characterized by irrational use of artesunate, or any other anti-malarials for patients without indications, are common and contribute to the vicious circle of medicine stock-outs and unnecessary costs of treatment. Beside the establishment of the effective supply chain, carefully tailored, multifaceted programmatic interventions combining educational, supervisory and group problem solving components accompanied with regular monitoring and feedback to health workers remain the quality improvement priority in the study area [12, 24].

With respect to artesunate dosing, most patients were correctly prescribed minimum of three doses however high likelihood of underdosing and incomplete anti-malarial treatments have been suggested. Prior studies found dose approximations for adults [12] while this assessment revealed similar practices across all age groups. Strikingly uniformed artesunate prescriptions of 120 mg for adults are unlikely to reflect 50 kg patients’ weight distribution but more likely cost considerations, minimizing of the waste and single dose rationing matching two vials of artesunate. While recently reported in Uganda [25], this study finding showing low levels of ACT follow-on treatment despite high ACT availability and high knowledge of the prescribers about this standard is the worrisome findings requiring further investigations behind the reasons for such practice which inevitably contributes to compromised cure rates [26].

Finally, the case-management practices reported here are based on data extraction from the non-structured routine records and admission medical files. While authors are confident about the documentation of the key malaria test and treatment data elements, incomplete documentation of the clinical features in admission files and subsequent misclassification of the malaria severity cannot be excluded. To mitigate these possibilities documented clinical severity features were complemented with health workers’ admission diagnosis of severe malaria. While such approach may benefit measurements of the health worker performance compared to the sole use of gold standard classifications, this limitation however, considering the scale of suboptimal practices described in the study, is unlikely to change the study conclusions. Nevertheless, introduction of the structured admission record forms to facilitate data collection and performance monitoring but also to serve as clinical job aid to improve quality of care [27, 28] can be considered in the study settings.

## Conclusions

The study revealed some strengths but also major systems readiness and clinical practice deficiencies which severely compromise the quality of service delivery for patients admitted with suspected malaria in Kano State in Nigeria. Establishments of the effective supply chain for the medicines, quality assured diagnostics, ongoing support for clinicians and nurses to deliver care according to the standards and close monitoring of the systems readiness and clinical practices will ultimately determine the success of the policy translation into practice and the quality of inpatient malaria case-management.

## Data Availability

The datasets used and analysed during the current study are available from the corresponding author on reasonable request.
